# Ecstasy-Induced Acute Myocardial Infarction Unraveled by Multimodality Imaging

**DOI:** 10.7759/cureus.86739

**Published:** 2025-06-25

**Authors:** Glenmore Lasam, Maria Kristina Cassandra Lasam, Loba Alam, Linus Nweke

**Affiliations:** 1 Cardiovascular Diseases, Stockton Cardiology Medical Group, Manteca, USA; 2 Biomedical Pathway, Mountain House High School, Mountain House, USA; 3 Internal Medicine, Overlook Medical Center, Summit, USA; 4 Internal Medicine, Northern Westchester Hospital/Northwell Health, Mount Kisco, USA

**Keywords:** acute myocardial infarction, cardiac magnetic resonance imaging, methylenedioxymethamphetamine, multimodality cardiac imaging, optical coherence tomography

## Abstract

A case of a 36-year-old male with no known comorbidities presented because of chest discomfort after using ecstasy and was noted to have an evolving anteroseptal myocardial infarction (MI). Left heart catheterization showed mild obstructive coronary disease with red thrombus in the proximal left anterior descending (LAD) artery seen on optical coherence tomography (OCT) and with note of improvement and decrease in thrombus burden on repeat coronary angiogram the next day after starting the patient on eptifibatide. Echocardiogram revealed mildly reduced systolic function with an apical akinesis. Cardiac magnetic resonance imaging (CMR) showed a small-sized infarct involving the apical inferior, apical lateral, and basal septum, with myocardial edema at the apex and septum, and a note of a small apical clot. He improved significantly during his course and was maintained on ticagrelor, apixaban, and atorvastatin.

## Introduction

3,4-Methyl​enedioxy​methamphetamine (MDMA), commonly known as ecstasy, is a psychoactive drug predominantly used for recreational purposes. It is a frequently abused drug, especially among young partygoers at dance clubs and festivals. Several dance celebrations in the United States have encountered partygoer deaths from ecstasy use [1]. When taken orally, desired effects begin within half an hour and can last several hours, which include feelings of heightened energy, wakefulness, outrage, intimacy, excitement, and disinhibition [2]. There were few reports in the literature regarding the cardiovascular sequela, including myocardial infarction (MI), which has been linked to its sympathomimetic effects. There is a paucity of published data linking MDMA to MI, with no reported prevalence in the literature since most of the cases were clinical vignettes. Multimodality imaging, which includes coronary angiography, optical coherence tomography (OCT), cardiac magnetic resonance (CMR), and echocardiography, is an essential tool to elucidate further the inciting etiology of MI, especially in young individuals presenting with an acute coronary event associated with MDMA use. The early utilization of such a method ushers in early diagnosis, which leads to appropriate and timely management.

This article was previously presented as a poster presentation at the American College of Cardiology 71st Annual Scientific Session on April 2, 2022, in Washington, District of Columbia, USA.

## Case presentation

A healthy 36-year-old male with no known comorbidities presented to the hospital because of chest discomfort. He described the discomfort as pressure-like, located in the mid-sternal region, persistent, exertional, severe in intensity, and associated with nausea, vomiting, and diaphoresis. He has never had chest discomfort of this magnitude in the past, nor has he dealt with issues of pericarditis, costochondritis, cardiopulmonary infections, or gastroesophageal reflux disease. He admitted that he had used ecstasy for the past six hours and danced at a house party at that time. He vehemently denied use of cocaine, heroin, and other illicit drugs but has some alcohol. He denies any recent febrile episodes or chills and has completed his COVID-19 vaccine series. He is not an active smoker. He has no significant family history of premature coronary artery disease or sudden cardiac death.

**Figure 1:**
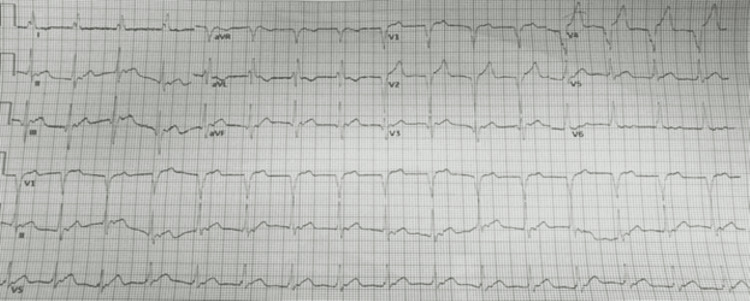
Initial electrocardiogram showing sinus rhythm, normal axis, and evolving ST wave elevation in the anteroseptal leads.

**Figure 2:**
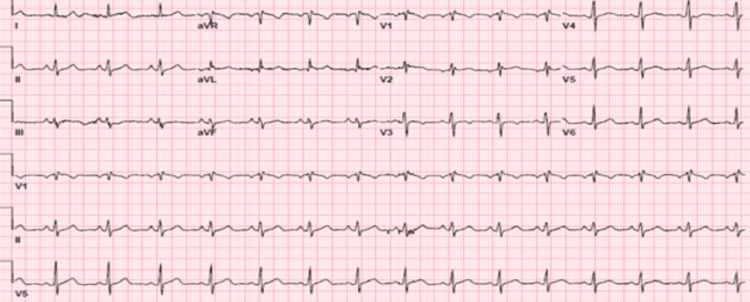
Post coronary angiogram electrocardiogram showing sinus rhythm, normal axis, and resolution of the acute ST wave changes in the anteroseptal leads.

**Figure 3:**
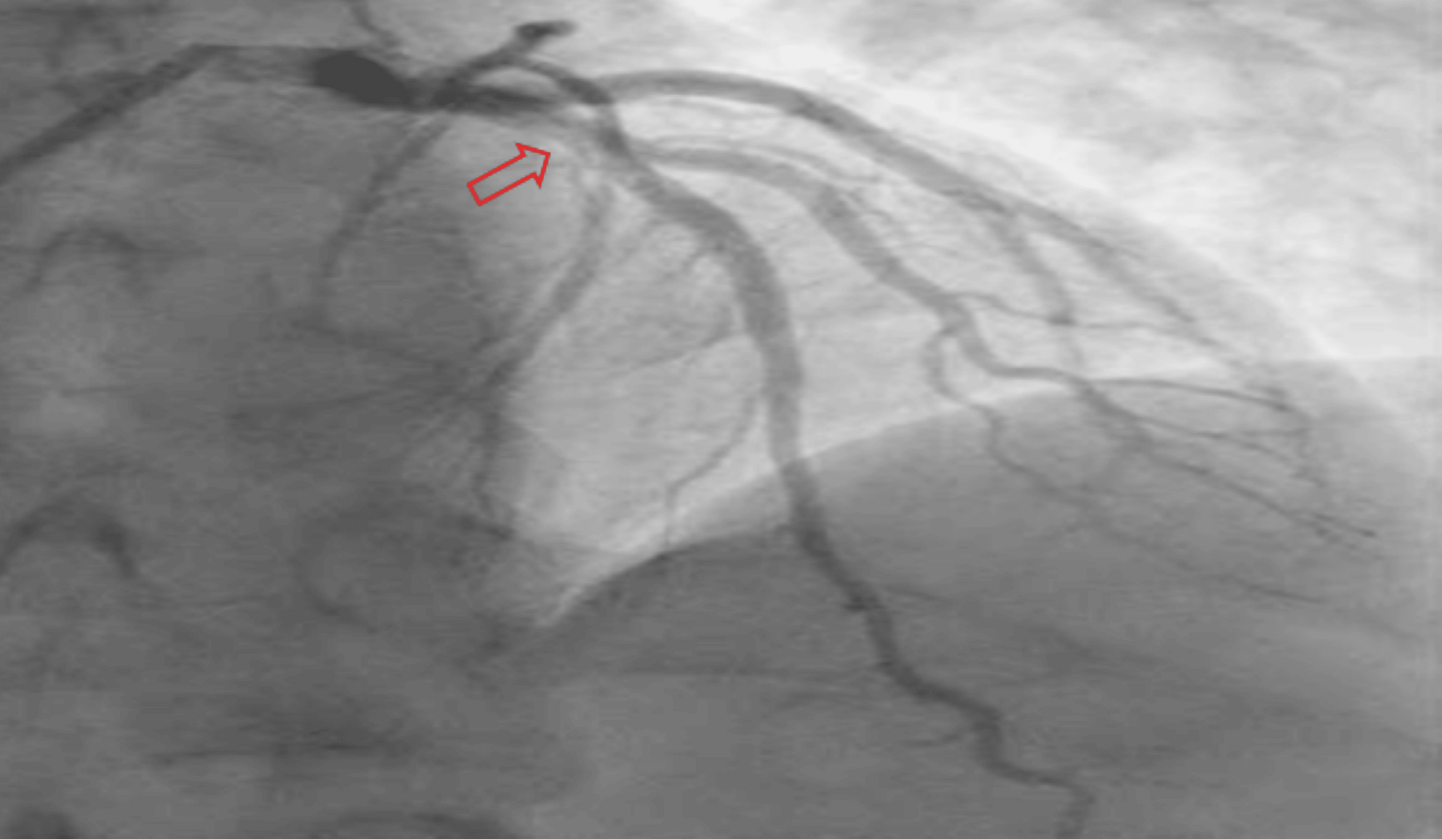
Initial coronary angiogram revealed thrombotic lesion in the proximal left anterior descending artery with 30-50% stenosis.

**Figure 4:**
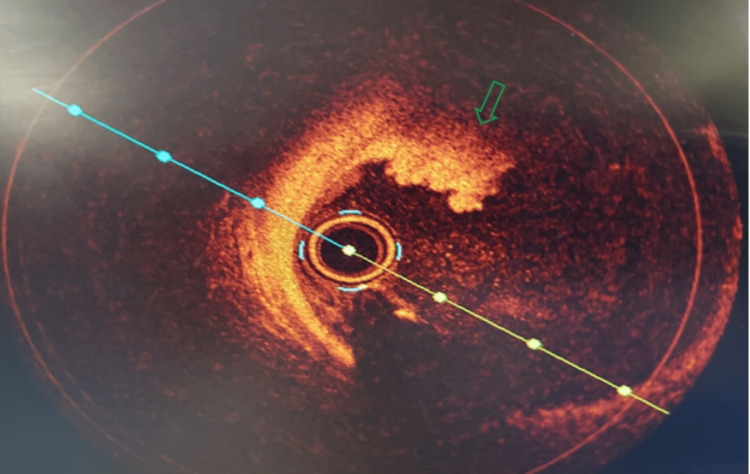
. Optical coherence tomography of the proximal left ascending coronary artery showed red thrombus with mild burden and with no atherosclerotic plaque.

**Figure 5:**
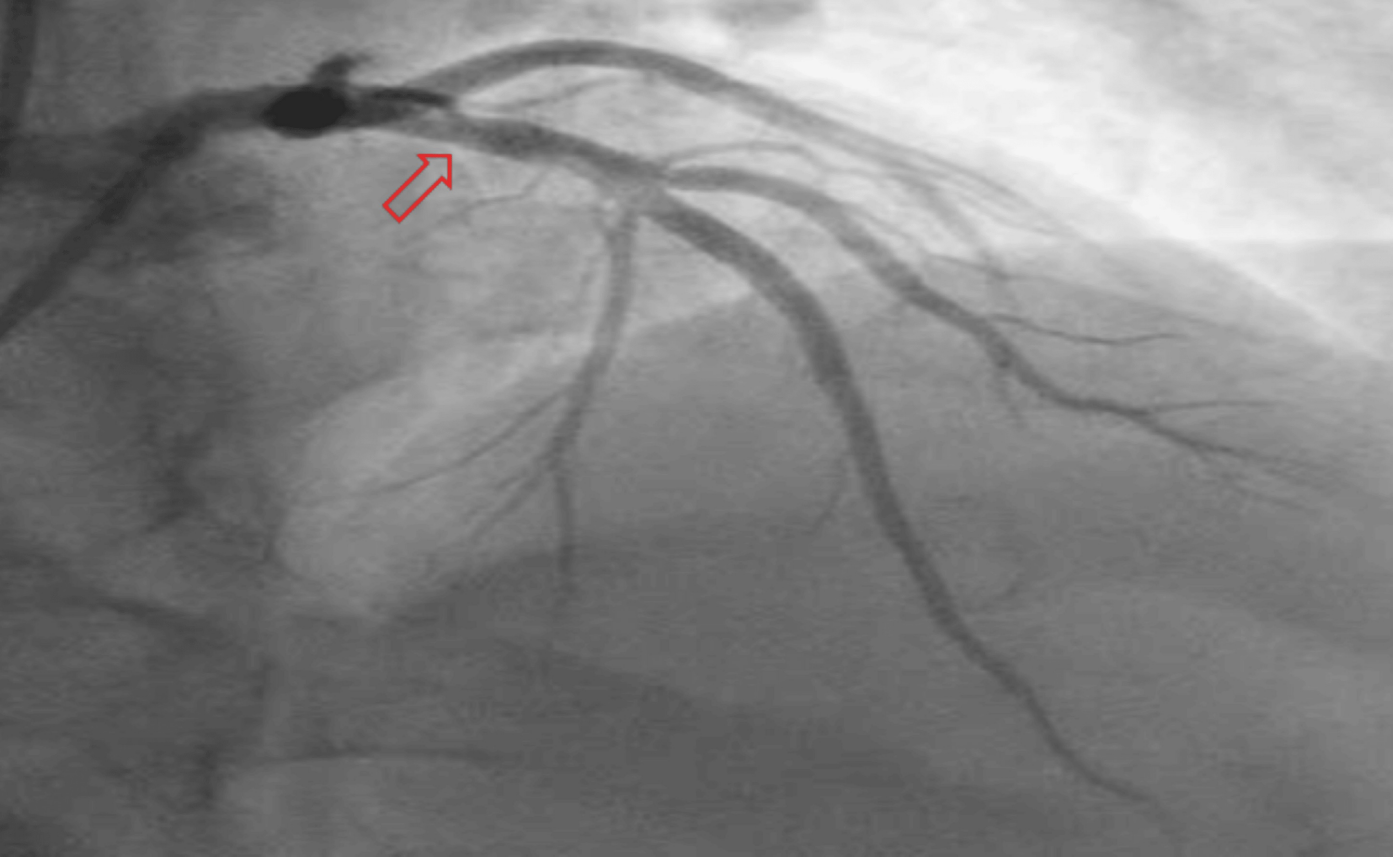
Repeat coronary angiogram revealed thrombotic lesion (less severe) in the proximal left anterior descending artery with <30% stenosis.

**Figure 6:**
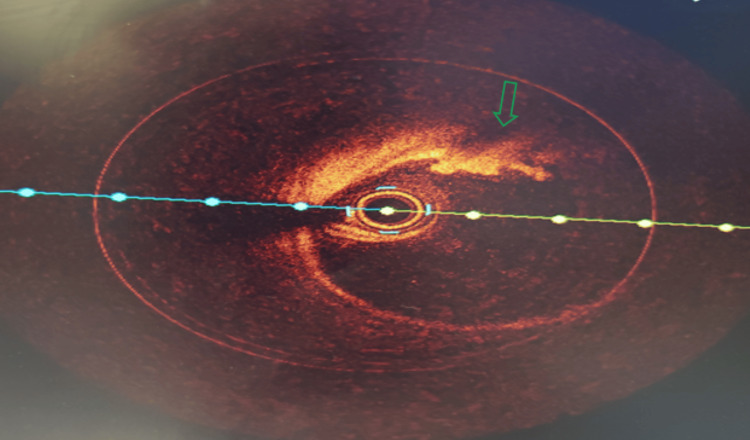
Optical coherence tomography (OCT) of the proximal left ascending coronary artery showed a decreased thrombus size (>50% from the initial OCT) with mild burden.

**Figure 7:**
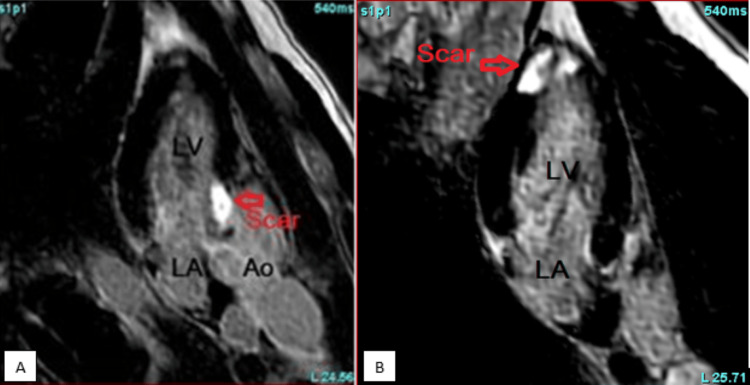
Cardiac magnetic resonance imaging showing transmural late gadolinium enhancement in the basal septum (A) and significant late gadolinium enhancement in the apical inferior and apical lateral region (B).

**Figure 8:**
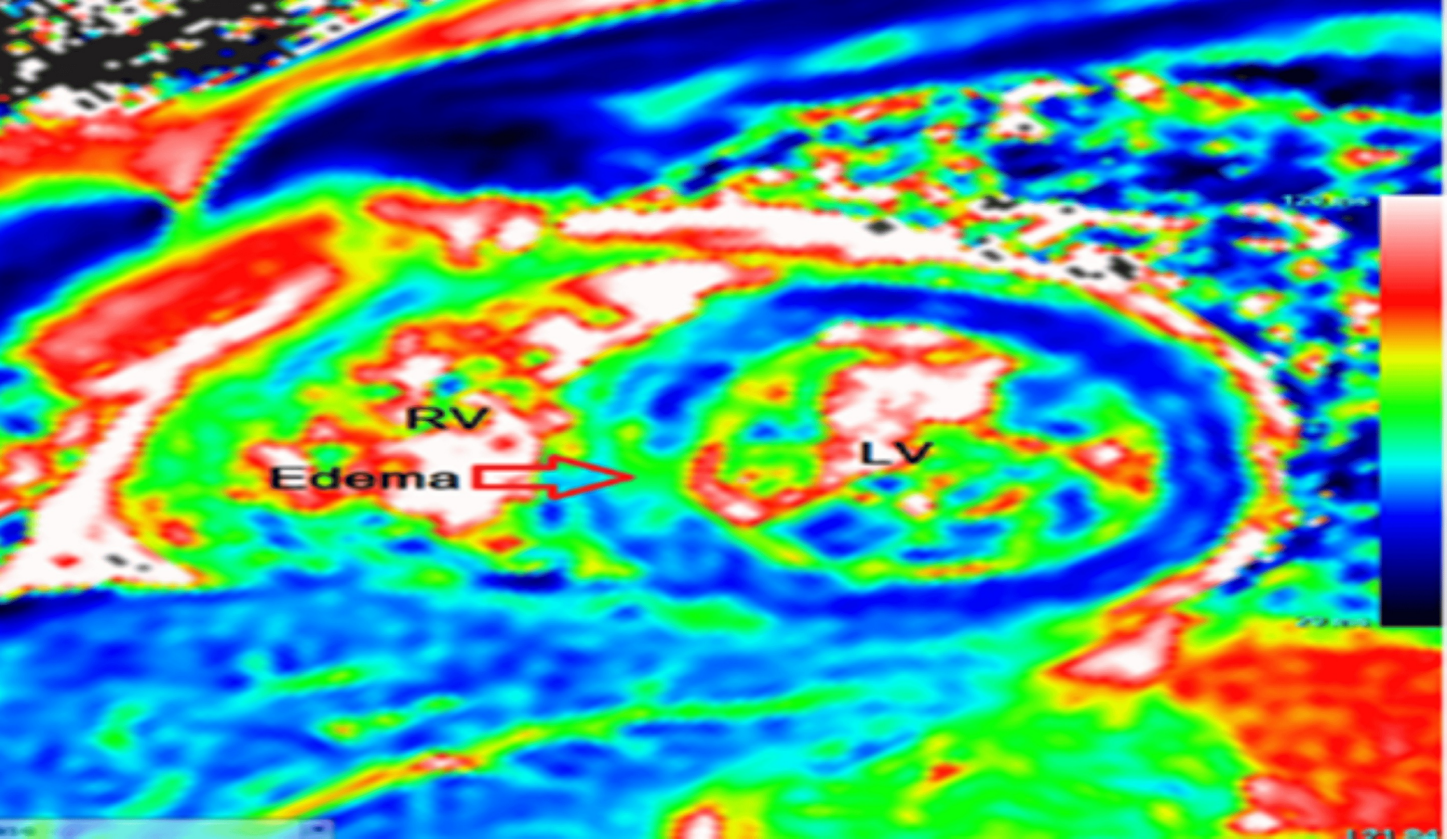
Cardiac magnetic resonance imaging utilizing T2 mapping demonstrating myocardial edema.

In the emergency room, he was found to be pale and diaphoretic but with a temperature of 98℉, blood pressure of 100/60 mmHg, heart rate of 100, respiratory rate of 18, and oxygen saturation of 99% on room air. Cardiac examination revealed a regular rhythm with no murmurs or gallop. Respiratory assessment elucidated clear breath sounds. Initial electrocardiogram showed sinus rhythm, normal axis, and evolving ST wave elevation in the anteroseptal leads (Figure 1). Troponin was mildly elevated at 2.016 ng/ml. Hemogram and lipid profile were unremarkable. C-reactive protein was 1.8 mg/L, and erythrocyte sedimentation rate was 19.2 mm/hr. Urine toxicology screening was remarkable for MDMA but was negative for methamphetamine and cocaine. Chest radiograph revealed no active cardiopulmonary disease. He underwent left heart catheterization, which revealed mild obstructive coronary disease (Figure 3) with a note of red thrombus in the proximal LAD artery seen on OCT (Figure 4). Electrocardiogram post-procedure showed sinus rhythm, normal axis, and resolution of the acute ST wave changes initially seen in the anteroseptal leads (Figure 2). He was started on eptifibatide and was admitted to the coronary care unit. A repeat left heart catheterization (Figure 5) with OCT (Figure 6) showed improvement and a decrease in thrombus burden in the proximal LAD artery with no atherosclerotic plaque. The echocardiogram showed mildly reduced systolic function with an ejection fraction of 50% and noted apical akinesis with swirling of contrast suggestive of stasis. CMR showed delayed myocardial enhancement indicative of a small-sized infarct involving the basal septum (Figure 7A), apical inferior, and apical lateral region (Figure 7B), with myocardial edema at the apex and septum (Figure 8), and a note of a small apical clot. He had a stable five-day course of hospitalization with no episodes of hemodynamic instability and with resolved chest discomfort after initiation of medical therapy. He was discharged improved and was maintained on ticagrelor, apixaban, and atorvastatin. He has been followed up as an outpatient within two weeks post-discharge and has been feeling better with no recurrence of chest discomfort. 

## Discussion

MDMA is a widely used illicit drug used by a large number of young people in nightclubs, and its effects on altering the mind and leaving users with extreme changes in body temperature and fluid balance have been well publicized. Like cocaine and other powerful sympathomimetics, it is understood that ecstasy can cause acute MI by inducing severe coronary vasospasm [3]. MDMA stimulates the release of noradrenaline (norepinephrine), dopamine, and serotonin from the central and autonomic nervous systems and has a monoamine oxidase inhibitor effect, inhibiting the reuptake of catecholamines in sympathetic synapses [4]. Its effects start about 20 minutes after intake and can last up to 48 hours later [5], as in the case of this patient. In addition, when combined with alcohol, similar to our vignette, there can be a potential sympathetic surge that can cause tachycardia, essential hypertension, arrhythmia, and MI. It is crucial to note that the precise mechanisms by which MDMA triggers prothrombotic effects are still being explored, and further research is required to completely recognize the risks. Several factors are thought to contribute including rhabdomyolysis and myosin release, which are potential trigger for coagulation as it can increase thrombin generation in plasma independently of platelets; hyperpyrexia, which increases levels of von Willebrand factor; endothelin; and intercellular adhesion molecule 1 (ICAM-1) that can activate the vascular endothelium that generate prothrombotic state; and oxidative stress and disruptions of tight junctions leading to endothelial damage, which increases blood vessel permeability and further potentially contributing to thrombosis [6]. The patient had an acute thrombus in the LAD territory, which predisposed to left ventricular apical akinesis and eventually developed left ventricular apical thrombus. The use of anticoagulation together with a P2Y12 receptor blocker in a patient with left ventricular thrombus in a recent acute MI is an acceptable regimen, but it is important to consider the potential for increased bleeding risk [7]. However, our patient is considered low risk for bleeding. In the medical literature, there was a reported case of MI in a teenager in 2010 [8]. Another occurrence was reported in a young man with acute MI shortly after taking ecstasy, and he was noted to have massive thrombosis on the right coronary artery, demonstrated by emergency coronary angiography [9]. It is noteworthy that in both cases, the patients did not present significant characteristic clinical risk factors for myocardial infarction. Although ecstasy-induced myocardial infarction is a rare entity, it can occur as documented in the literature.

This case highlights the importance of obtaining necessary drug history in young patients who present with chest pain. As is often the case, clinical management is of the essence. The chest pain that patients often present with can be due to coronary vasospasm, and although there is no evidence-based approach in the medical literature, the appropriate treatment is usually pharmacological. The patient was young and had no evidence of recent cardiopulmonary infection nor hyperacidity symptoms, which unlikely consider myopericarditis, costochondritis, and gastroesophageal reflux disease as possible etiologies of his chest discomfort. On coronary angiogram, there was no note of spontaneous coronary artery dissection or coronary artery vasospasm. Of importance, the avoidance of beta blockers is essential in patients with stimulant intoxication because it may aggravate hypertension and coronary artery vasospasm due to loss of beta (2)-mediated vasodilation and unopposed alpha-receptor activation [10].

As seen in our case, coronary angiography with the use of OCT can make a difference as it can be used to establish coronary artery disease or the presence of an occlusive thrombus, and with that, the use of an antiplatelet agent to stabilize or decrease the clot burden can be justified. In this vignette, MDMA is temporally associated with MI, although causality remains presumptive based on circumstantial and imaging findings. OCT and CMR complemented each other to rule out the thrombus and infarction, respectively, in this case. OCT plays an important role in acute MI through its high spatial resolution that allows for precise evaluation of plaque characteristics, providing detailed insights into culprit lesions, thrombus identification, guiding percutaneous coronary intervention, aiding in lesion preparation for stent deployment optimization, and visualization of procedural complications including malapposition, tissue prolapse, and dissections [11]. CMR provides highly reproducible and accurate evaluation of cardiac function and detailed characterization of myocardial tissue in the assessment of acute MI, providing precise information about the degree of myocardial insult, including infarct size, viability, and the presence of microvascular obstruction [12]. CMR also assists in assessing risk stratification, anticipating long-term outcomes, and directing therapeutic decisions [13]. The CMR findings in this vignette are consistent with infarction and not with other etiologies of chest discomfort in young patients, including pericarditis or myocarditis. The use of CMR, as in this case, can help in ruling out other potential causes from a structural myocardial standpoint while also assessing for myocardial function. In our presented case, CMR was performed to determine the extent of myocardial scar. Transmural myocardial infarction was seen at the base of the septum and at the apex, with myocardial edema on T2-mapping, indicating acute myocardial injury. Acute MI in multiple territories, an apical clot, and a red thrombus on OCT were all indicative of a hypercoagulable state possibly linked to MDMA use. The imaging findings guided the long-term therapy for antiplatelet and anticoagulation until the left apical thrombus resolves, and maintenance solely on antiplatelet thereafter. Primary hypercoagulability state has been excluded eventually with unremarkable investigative tests, including factor V Leiden mutation, prothrombin gene mutation, antithrombin III, protein C, and protein S. Also, an echocardiogram is indispensable in this case, which offers valuable information for diagnosis, risk stratification, and treatment guidance through the assessment of regional wall motion abnormalities, infarct size, and detection of potential complications [14]. 

## Conclusions

This vignette emphasizes the benefit of combining coronary angiography with other imaging modalities to determine the etiology of MI in patients who have no substantial atherosclerosis. The therapy has been tailored based on imaging findings of the presence of coronary vessel thrombus and infarction through the use of antiplatelet (GP IIb/IIIa receptor blocker). A direct-acting oral anticoagulant was added to the regimen because of the finding of apical thrombus on echocardiogram. MDMA is temporally associated with MI in this case, although the connection remains presumptive based on multimodal imaging results. Clinicians should consider MDMA as an etiology of MI, albeit more research on stimulant-related MI in young patients should be conducted to elucidate more confirmatory causality. 
